# Hypoxia-Induced Retinal Neovascularization in Zebrafish Embryos: A Potential Model of Retinopathy of Prematurity

**DOI:** 10.1371/journal.pone.0126750

**Published:** 2015-05-15

**Authors:** Yu-Ching Wu, Chao-Yuan Chang, Alex Kao, Brian Hsi, Shwu-Huey Lee, Yau-Hung Chen, I-Jong Wang

**Affiliations:** 1 Department of Ophthalmology, National Taiwan University Hospital, Taipei, Taiwan; 2 Department of Chemistry, Tamkang University, Tamsui, New Taipei City, Taiwan; 3 Department of Ophthalmology, Cathay General Hospital, Taipei, Taiwan; Purdue University, UNITED STATES

## Abstract

Retinopathy of prematurity, formerly known as a retrolental fibroplasia, is a leading cause of infantile blindness worldwide. Retinopathy of prematurity is caused by the failure of central retinal vessels to reach the retinal periphery, creating a nonperfused peripheral retina, resulting in retinal hypoxia, neovascularization, vitreous hemorrhage, vitreoretinal fibrosis, and loss of vision. We established a potential retinopathy of prematurity model by using a green fluorescent vascular endothelium zebrafish transgenic line treated with cobalt chloride (a hypoxia-inducing agent), followed by GS4012 (a vascular endothelial growth factor inducer) at 24 hours postfertilization, and observed that the number of vascular branches and sprouts significantly increased in the central retinal vascular trunks 2–4 days after treatment. We created an angiography method by using tetramethylrhodamine dextran, which exhibited severe vascular leakage through the vessel wall into the surrounding retinal tissues. The quantification of mRNA extracted from the heads of the larvae by using real-time quantitative polymerase chain reaction revealed a twofold increase in *vegfaa* and *vegfr2* expression compared with the control group, indicating increased vascular endothelial growth factor signaling in the hypoxic condition. In addition, we demonstrated that the hypoxic insult could be effectively rescued by several antivascular endothelial growth factor agents such as SU5416, bevacizumab, and ranibizumab. In conclusion, we provide a simple, highly reproducible, and clinically relevant retinopathy of prematurity model based on zebrafish embryos; this model may serve as a useful platform for clarifying the mechanisms of human retinopathy of prematurity and its progression.

## Introduction

Retinopathy of prematurity (ROP), formerly known as retrolental fibroplasia [1], is one of the most common causes of infantile blindness [2] and is characterized by a vasoproliferative and fibrotic change in the vitreous body and retina [3]. In the Early Treatment for Retinopathy of Prematurity Study in the United States, the incidence of ROP among infants with a birth weight of less than 1251g was 68% and increased as the birth weight decreased [4].

Normal vascularization of the retina and vitreous body begins at approximately 16 weeks of gestation, radiating anteriorly from the optic nerve, and vascularization in the nasal and temporal retina is complete by 36 and 40 weeks, respectively; at this point, vascularization is sufficient to support retinal activity at birth [5, 6]. In ROP, prematurity leads to incomplete retinal vascularization in the early gestational age. Two phases of ROP can be distinctively identified, namely, an initial phase of vessel loss followed by a second phase of vessel proliferation [7]. First, an acute phase in which the hyperoxic extrauterine environment supplements the underdeveloped lung causes vasoconstriction and vasoattenuation of the remaining vascular growth through apoptosis [8]. The avascularized retina in ROP becomes increasingly hypoxic with metabolic activity and growth. This leads to the second chronic phase of ROP, which involves rapid neovascularization with hypoxia and the expression of hypoxia-inducible transcription factor (HIF) and vascular endothelial growth factor (VEGF) [8]. The second phase progresses as a highly disorganized fibrovascular proliferation from the demarcated ridge along the retina. As the severity increases, partial and eventual total retinal detachment occur [9].

Regarding ROP pathogenesis, VEGF appears to play a critical role in both normal physiological and pathological angiogenesis [10–14]. VEGF is highly regulated by hypoxia through HIF-1α and HIF-2α which can react with hypoxia response elements and induce transcriptional activity [15]. Therefore, hypoxia causes the second phase of ROP. Numerous studies have recognized that cobalt chloride (CoCl_2_) promotes a response similar to hypoxia [16] because cobalt iron can replace iron from the iron-binding center of specific prolyl hydroxylases and inactivate hydroxylation activity [17]. In addition, CoCl_2_ directly binds to HIF-1α and causes HIF-1α accumulation by inhibiting its binding to the von Hippel-Lindau protein, a mediator of HIF-1α degradation; moreover, CoCl_2_ elicits hypoxic conditions [18]. Chemical hypoxia agents have been widely used in numerous systems [19–22] because it is inexpensive and easy to control the level of hypoxia by varying the concentration.

Animal models of ROP have yielded much of the current knowledge on physiological and pathological blood vessel growth in the retina. However, animal models of oxygen-induced retinopathy have limitations because the animals are not always premature. Nonetheless, these models have substantially enhanced the understanding of ROP pathogenesis [23–25]. For example, the rat model of ROP consistently produces a robust pattern of retinal neovascularization similar to that observed in humans [24]. However, this model has drawbacks, namely strain- and vendor-related differences in susceptibility to neovascularization, a substantial amount of time required to yield a result, and insufficient cost effectiveness. Furthermore, the mouse model has generated different vascular growth patterns when subjected to the same conditions that induce ROP [26].

Zebrafish (*Danio rerio*) models are superior to other animal models because of their transparency, which facilitates *in vivo* observation; low cost; practicality; and high fecundity. Numerous similarities in the retinal vasculature and cellular hallmarks to humans enable the zebrafish embryo to model retinal neovascularization and ROP [27–29]. Because ROP is a developmental disease, zebrafish embryos provide a model for rapidly evaluating effects and therapeutic treatments with a large sample size in a short time frame [30].

We established an ROP model in the Tg(*fli1a*:*EGFP*) transgenic zebrafish line by using CoCl_2_ as a hypoxia-inducing agent and a VEGF-inducer, GS4012, as a vasoproliferative agent to induce the two phases of ROP. Treating zebrafish with CoCl_2_ significantly increased the number of vascular branches and sprouts in the central retinal vascular trunks, reflecting stage 3 of human ROP, 2–4 days after treatment [23]. In addition, we employed an angiography method that involves using tetramethylrhodamine (TAMRA) dextran, which exhibited severe vascular leakage through the vessel wall into the surrounding retinal tissue. Finally, the pathologies were validated using anti-VEGF monoclonal antibodies, bevacizumab, and ranibizumab, similar to those treatments in clinical scenarios.

## Materials and Methods

### Zebrafish lines

All animal protocols were approved by the National Taiwan University Hospital Institutional Animal Care and Use Committee (IACUC) and conducted in accordance with the ARVO Statement for the Use of Animals in Ophthalmic and Vision Research. A transgenic zebrafish line, Tg(*fli1a*:*EGFP*), was obtained from the Zebrafish International Resource Center, and adult zebrafish were maintained in tap water at 28.5°C. The mating and spawning of zebrafish were incited by the change of dark to light. Embryos were collected at 1 to 6 days postfertilization (dpf) for observation in this study.

### CoCl_2_ and GS4012 treatments

To establish chemically induced hypoxia and neovascularization, cobalt (II) chloride hexahydrate (CoCl_2_.6H_2_O; Sigma-Aldrich, St. Louis, Missouri, United States) and a VEGF-inducer GS4012 (Merck K GaA, Darmstadt, Germany) were used to stimulate abnormal retinal angiogenesis. A preliminary dose and time dependence response study was conducted to determine the optimal conditions for phenotype observation. Embryos were treated with CoCl_2_ (1–20 mM) or GS4012 (2.5–7.5 μg/mL)[31] at 1 dpf, and their effects were observed and statistically analyzed at 3 and 5 dpf. Because of the marked vascular defects with low mortality observed at 5 mM CoCl_2_ or 2.5 μg/mL GS4012, we used these concentrations in subsequent experiments.

### Quantitative real-time RT-PCR

A real-time PCR study that involved using zebrafish treated with 5 and 10 mM CoCl_2_ was conducted to confirm that CoCl_2_ can induce hypoxia through our proposed mechanism of *vegfaa*, *plxnd1*, and downstream targets *vegfr2*. Total RNAs were extracted from the heads of 50–70 zebrafish embryos by using the TriSolution Reagent (GMbiolab). The RNA pellets were washed with cold 70% ethanol and dissolved in DEPC-treated water. Reverse transcription was conducted using Go-Script reverse transcriptase (Promega, Madison, Wisconsin, United States) and total RNA (2–4 μg) was used to synthesize cDNA by using an oligo-dT primer (MBBioInc). To verify the gene expression of *vegfaa* and *vegfr2*, quantitative real-time RT-PCR (qRT-PCR) was performed using the SYBR-Green fluorescence label (Applied Biosystems, Life Technologies, Carlsbad, California, United States). Each PCR reaction mixture contained 10 μL of the SYBR-Green label, 5 μL of cDNA, and 2 μL of specifically designed primer sequences at 2 μM (Table 1). The cycling conditions are described as follows: 95°C for 3 minutes; 40 cycles at 95°C for 10 seconds and the optimal primer temperature for 45 seconds; 95°C for 1 minute; and 65°C for 1 minute. β-*actin* was used as the internal control gene for reference, and mRNA levels were standardized against it. All reactions were performed in triplicate on cDNA isolated from three independent experiments.

**Table 1:** qPCR primer sequences.

*vegfaa*-forward	ACCCCCTCACCTGTAAATGCT
*vegfaa*-reverse	AGTTGTCTGGACTTGCATTGAGT
*vegfr2*-forward	CCATCGAACCAGAAAGACCAAG
*vegfr2*-reverse	ACGATTGATCCGCTCCTTATGA
*plxnd1*-forward	ACAGATCCGCGACGAGATAC
*plxnd1*-reverse	CTCTTTGGTCAGATCCGTCAT
*β-actin*-forward	CAGCAAGCAGGAGTACGATGAGT
*β-actin*-reverse	TTGAATCTCATTGCTAGGCCATT

### Anti-VEGF treatments

Three VEGF inhibitors, SU5416 (Merck KGaA, Darmstadt, Germany), bevacizumab (Avastin, Roche, Basel, Switzerland), and ranibizumab (Lucentis, Novatris, Stein Switzerland) were used as candidate drugs to reverse abnormal retinal neovascularization. SU5416 targets the VEGF receptor 2 tyrosine kinase and is a potent, selective competitive inhibitor of Flk-1/KDR [1]. Bevacizumab is a humanized monoclonal antibody that is derived from antibody A4.6.1 [32] and binds to membrane-bound receptors (VEGF receptors-1 and -2) [33, 34]. Bevacizumab blocks VEGF receptor binding and signaling; its epitope overlaps the epitopes of VEGF receptors-1 and -2. Similarly, ranibizumab [35] is a recombinant humanized IgG1 kappa isotype monoclonal antibody fragment with a high binding affinity for all forms of VEGF. All of these inhibitors have been shown to inhibit angiogenesis [36, 37]. Zebrafish were treated with 0.5 μM SU5416 [38], 2.5 μg/mL of bevacizumab [39], or 2.5 μg/mL of ranibizumab as previously described to examine the reverse efficiency of neovascularization in the current model. Furthermore, 2.5 μg/mL of normal human control IgG (R&D system, Minneapolis, MN) was used as a control.

### Fluorescent dye injections

Embryos were anesthetized prior to dye injection. Tg(*fli1a*:*EGFP*) fish that were or were not treated with CoCl_2_ at 3 dpf and 5 dpf were injected with 2.5–10 mg/mL of 10,000 MW Dextran, Alexa Fluor 546 (Invitrogen, Carlsbad, California, United States). The fish were subsequently observed under the LSM 780 confocal microscope (Carl Zeiss, Oberkochen, Germany) to identify disorganized vascular architecture and vessel leakage at 3- and 9-minute marks on Day 3 postfertilization and 10- and 15-minute interval marks on Day 5 postfertilization. We used another dye, 2,000,000 MW TAMRA (Invitrogen, Carlsbad, California, United States) and compared its efficiency with 10,000 MW Dextran. The TAMRA dye at 2.5–10 mg/mL was injected upstream into the cardiac atria of the embryos at 4 dpf. At approximately 24 hours postinjection, 3- and 5-dpf embryos were fixed on a cover slip by using 5% methyl cellulose (Sigma-Aldrich, St. Louis, Missouri, United States) and examined under the confocal microscope (LSM 780, Carl Zeiss, Oberkochen, Germany) to identify changes in the disorganized vascular architecture and retinal vascular leakage induced by CoCl_2_ effected by candidate drugs.

### *In vivo* imaging

Our in vivo imaging methods were adopted and modified from those of Hartsock and Alvarez [27, 29]. One hour prior to imaging, embryos were anesthetized in 0.0015 M tricaine in fish water. We submerged 1–3-dpf embryos into 0.0015 M tricaine in 1% low-melt agarose (LMA; UltraClean Agarose LM; #15005) and submerged 3-dpf and older embryos into 0.0015 M tricaine in 1.2% LMA. The embryos were mounted in a glass-bottomed imaging dish immediately below the LMA surface for imaging on an upright microscope equipped with immersion objectives and at the glass surface for imaging on an inverted microscope. After 5 minutes at room temperature, the mounted embryos were completely submerged in fish water with tricaine (0.0015 M) and imaged using the inverted confocal microscope (LSM 780, Carl Zeiss, Oberkochen, Germany) under a 40× objective. Approximately 50–100 1-μm optical slices were acquired every 10–15 min. Each stack was compressed to a maximal projection by using Zeiss LSM software.

### Statistical analysis

Each *in vivo* experiment was conducted a minimum of three times. Dot plots contained data from one representative experiment from at least three biological replicates. Statistical analysis was performed using Graph Pad Prism V6.0. Data are presented as the means ± SD. Under the assumption that the overall population of values conforms to a Gaussian distribution, the differences between the means were tested for significance by using the nonparametric Mann Whitney U-test. A difference between two means was considered to be significant when *p* < 0.05. The Bonferroni correction was used to counteract the problem of multiple comparisons if the compared groups were more than 3 groups. The P values are included in the figure legends for each experiment.

## Results

### CoCl_2_ induced vasoattenuation in zebrafish embryos

**Fig 1:**
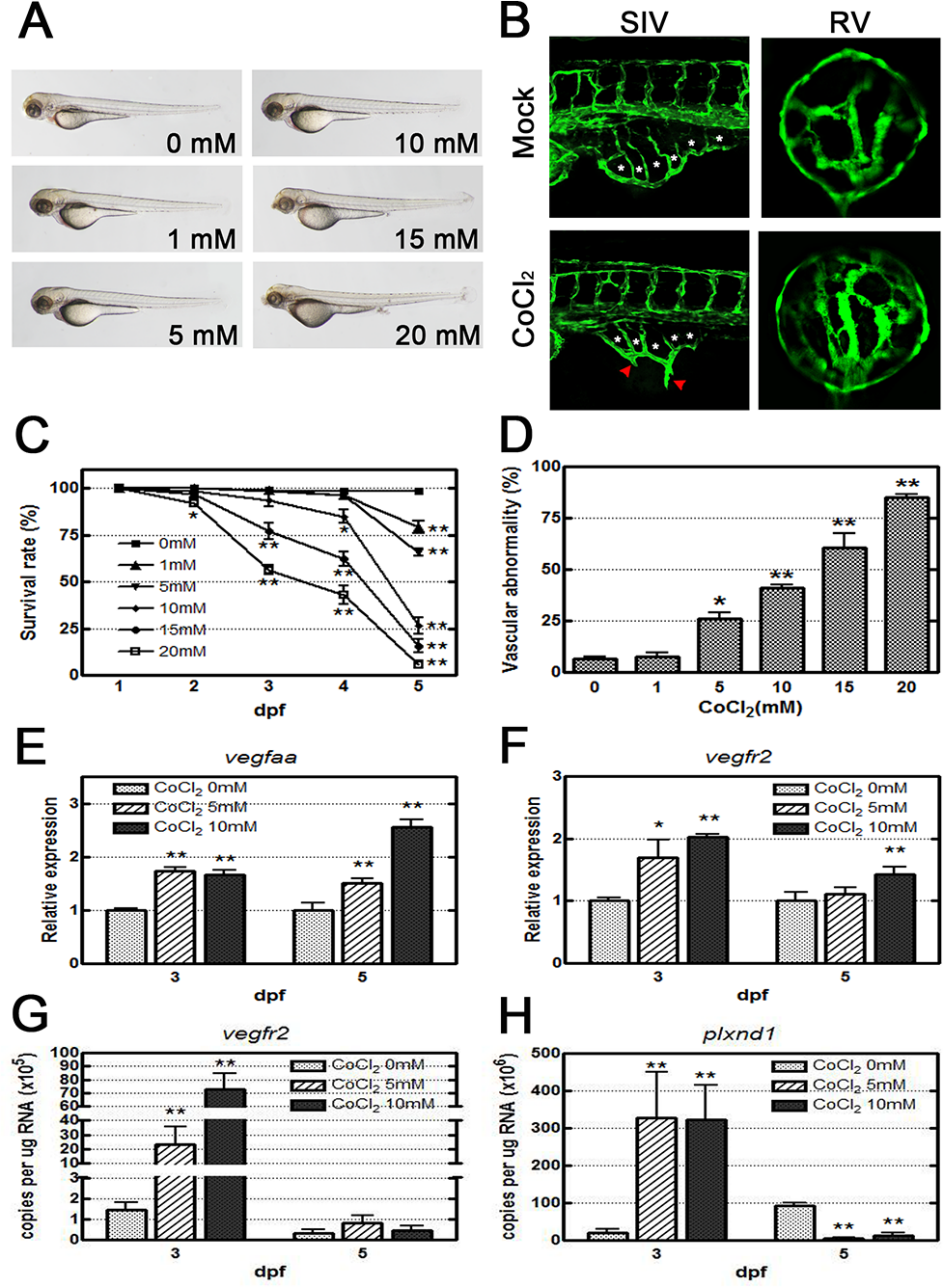
CoCl_2_ induces abnormal neovascularization in zebrafish embryos. (A) Morphological images obtained by an optical microscope revealed no severe phenotype in CoCl_**2**_-treated Tg(*fli1a*:*EGFP*) embryos at 3 dpf. (B) With fluorescence excitation, ectopic SIV and excessive retinal vascularization are shown in CoCl_**2**_-treated embryos compared with the untreated control. (C, D) A dose-dependent decrease in survival rates (four independent experiments; n = 35 in each group) in embryos treated with increasing concentrations (0–20 mM) and an increase in the vascular defect occurrence rate in the SIV and retinal vessels are shown (three independent experiments; n = 35 in each group). (E, F) Real-time RT-PCR data show that CoCl_**2**_ treatment causes overexpression of *vegfaa* and *vegfr2* mRNAs in zebrafish. (G,H) Absolute quantification of the copy number of *vegfr2* and *plxnd1* by real-time PCR are reduced at 5 dpf versus 3 dpf. Each bar represents the mean ± SEM. * (*p* < 0.01) and ** (*p* < 0.001) compared with the mock control group. SIV, subintestinal vessel; RV, retinal vessel.

Tg(*fli1a*:*EGFP*) embryos treated with CoCl_2_ exhibited no obvious morphological phenotype (Fig 1A). However, under a fluorescent microscope, they showed prominent vasoconstriction and vasoattenuation in the subintestinal vessel (SIV) plexus and retinal vessels (Fig 1B). The SIV appeared as a smooth basket-like structure with 6–7 arcades (asterisks) in the mock control (without CoCl_2_ treatment) group (Fig 1B), whereas ectopic vessels extruded as spikes (red arrowhead) ventrally from the SIV basket in the CoCl_2_-treated group at 3 dpf (Fig 1B). The CoCl_2_-treated embryos exhibited an increased number of branch points and sprouts in the retinal vessels compared with the mock control (Fig 1B). To investigate the dose effect of CoCl_2,_ the embryos were exposed to various concentrations (1–20 mM), and the dose-dependent effects of CoCl_2_ on the survival and occurrence rates of vascular defects in treated embryos were observed (Fig 1C and 1D). The results indicated that 5 mM CoCl_2_ was the optimal concentration because it provided sufficient stress to the fish with high survival rates (approximately 100% at 3 dpf and approximately 70% at 5 dpf). A qRT-PCR study was performed to quantify the mRNA levels of *vegfaa* and *vegfr*2 and, thus, confirm that CoCl_2_ induces hypoxia. The levels of *vegfaa* and *vegfr*2 from the heads of CoCl_2_-treated embryos were measured at 3 dpf and 5 dpf. Compared with the experimental controls (0 mM CoCl_2_), *vegfaa* and *vegf*r2 expression significantly increased approximately twofold at 3 dpf in the CoCl_2_-treated embryos, and the range of increase in *vegfr*2 decreased at 5 dpf (Fig 1E and 1F). Overexpression of *vegfaa* and *vegf*r2 mRNA is consistent with hypoxia, indicating that CoCl_2_-induced hypoxia in zebrafish is suitable for ROP disease modeling.

To further confirm the decrease of *vegfr2* mRNA in the CoCl_2_-treated embryos at 5 dpf and 3 dpf, we measured the copy number of *vegfr2* mRNA in the CoCl_2_-treated embryos. The result has been known that the absolute level of *vegfr2* mRNA at 5 dpf was 10-fold lower than that at 3 dpf (Fig 1G). Because the activation of Sema3E–Plexin-D1 signaling in tip cell of neovascularization has been know to regulate the VEGFR expression in stalk cell through Notch cascade [40], we examined the absolute level of *plxnd1* mRNA. Compared with the experimental controls, the copy number of *plxnd1* in the CoCl_2_-treated embryos increased in 10-fold at 3 dpf. In contrast, the copy number decreased approximately 10-fold reduced at 5 dpf (Fig 1H). These results imply that the decrease in *vegfr2* expression in the CoCl_2_-treated embryos at 5 dpf might result from the change of *plxnd1* signaling.

### Confocal analysis of retinal neovascularization under chemical hypoxia

To orient the images, we used a series of retinal tissues at each development stage over the first 6 dpf for reference (S1 Fig). The retinal vasculature sits in a hollow bowl-shaped structure in the center of the retina. For our model, we focused on the retinal vessels that branched from the central retinal artery in the zebrafish embryos (S1 Fig). At 1 and 2 dpf, normal vascular growth begins with vessel budding from the central retinal artery, which is separated from the annular collection duct. This vascular growth expanded over the following days, exhibiting signs of vessel branching at 3 and 4 dpf, forming the retinal vessels. Observing three to six main vessel branches, with each individual branch appearing relatively uniform and nontortuous, is relatively common; these branches form an organized vascular network.

**Fig 2:**
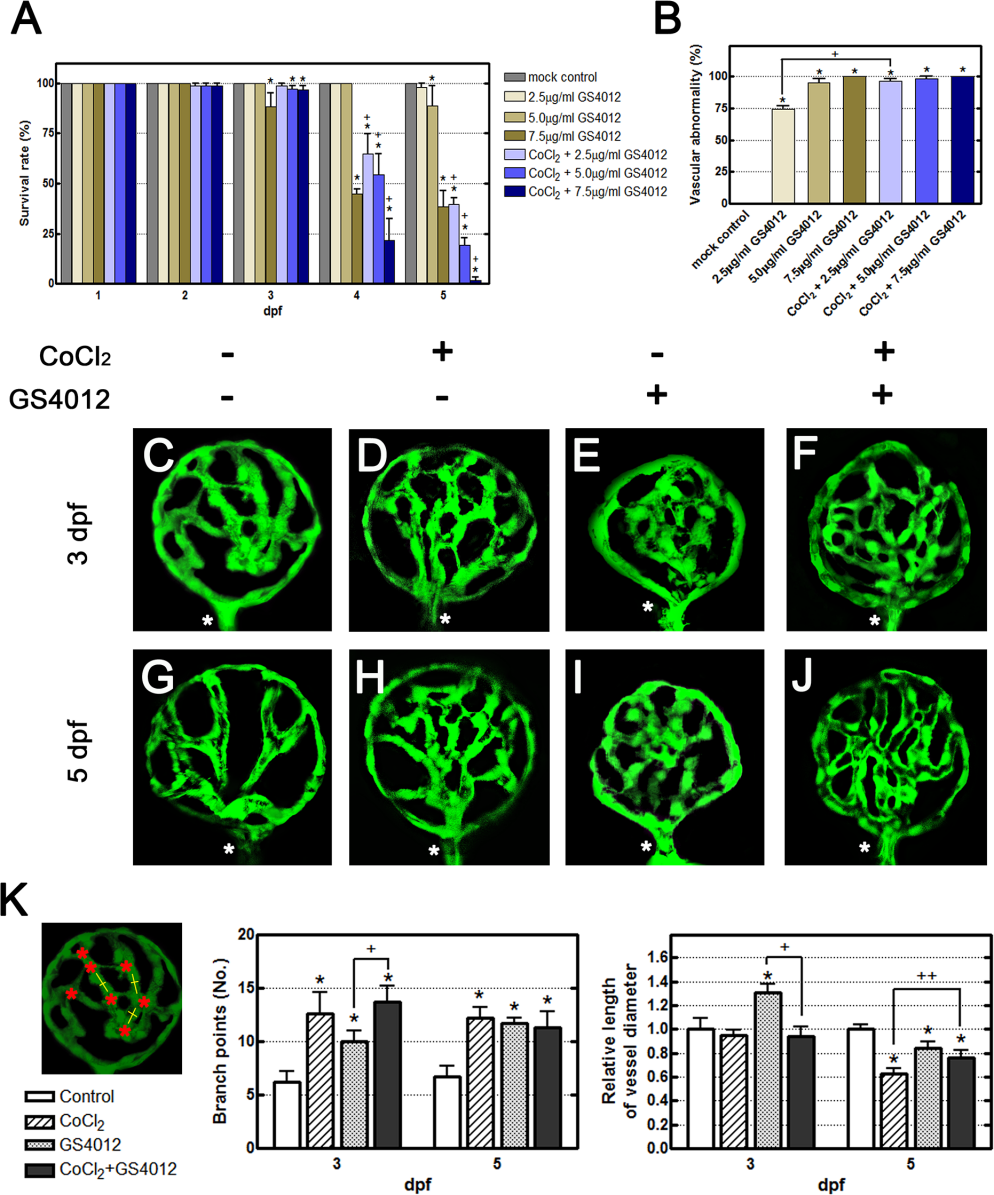
Effects of CoCl_2_ and the VEGF inducer GS4012 on retinal neovascularization. (A, B) Time-course and dose-dependent effects of GS4012 on the survival rate and the vascular defect-occurrence rates of Tg(*fli1a*:*EGFP*) embryos are shown. Embryos were treated with 2.5, 5, or 7.5 μg/mL of GS4012 and 5 mM CoCl_**2**_ for 1, 2, 3, 4, and 5 dpf. Each bar of the survival rate and the vascular defect-occurrence rates represents the mean ± SEM (n = 50 in each group). * *p* < 0.01 and + *p* < 0.001, as compared with the control group and the equivalent concentration of GS4012 group, respectively. (C–J) Fluorescence microscope observations of the retinal vessels of treated Tg(*fli1a*:*EGFP*) embryos are shown. At 3 dpf, compared with the untreated control (C), both CoCl_**2**_ and GS4012 induced vessel branching in the retina (D, E). Zebrafish embryos cotreated with CoCl_**2**_ and GS4012 showed severe branching and disorganization in the retinal vasculature (F). At 5 dpf, compared with the untreated control (G), CoCl_**2**_-treated retinal vessels were narrow, indicating vasoconstriction (H), and GS4012-treated vessels appeared tortuous and twisted (I). Furthermore, cotreatment with CoCl_**2**_ and GS4012 induced a complex, highly disorganized, and tortuous vasculature (J). (K) The left panel shows the method for counting and measuring branch points (red asterisks) and vessel diameters (yellow line). The vessel diameters were measured using Image J (three randomly chosen positions). These data consist of observed characteristics. Each bar of the branch points and vessel diameter chart represents the mean ± S.D. (n = 5 in each group). * *p* < 0.01, as compared with the control group. + *p* < 0.01 and ++ *p* < 0.001, as cotreatment groups compared with the equivalent concentration of GS4012 and CoCl_**2**_ group, respectively.

To examine retinal neovascularization, hypoxia was induced chemically by using CoCl_2_ in our pathology model, and GS4012 was used as the positive control. The observed effects confirmed the predictions from VEGF-induced pathway simulations. We expected a combination of CoCl_2_ and GS4012 to produce Loewe additivity to ensure the CoCl_2_-induced cascade. The survival and defect rates showed Loewe additivity as an additional CoCl_2_ was added with GS4012 (Fig 2A and 2B). The optimal doses of CoCl_2_ and GS4012 were adjusted according to the survival rates of embryos and the severity of morphological defects. The embryos cotreated with 5 mM CoCl_2_ and 2.5 μg/mL of GS4012 attained a 40% survival rate at 5 dpf, and all of the surviving fish exhibited SIV and retinal neovascularization (Fig 2A and 2B). A confocal examination of CoCl_2_- and GS4012-treated zebrafish revealed a disorganized and complex retinal vasculature (Fig 2C–2J). Treatment with 5 mM CoCl_2_ alone yielded an increased number of branch points and sprouts compared with that in the mock control group at 3 dpf (Fig 2C and 2D). At 5 dpf, the branch points were extensive and vessels narrowed, suggesting vasoconstriction (Fig 2G and 2H). This is consistent with the early characteristics of ROP (stage 3 of human ROP). Embryos treated only with GS4012 also showed signs of increased vessel branching at both 3 dpf and 5 dpf (Fig 2E and 2I). Comparing the cotreated CoCl_2_ and GS4012 zebrafish with the mock controls revealed apparent characteristics of both individual treatments previously mentioned. Increased tortuosity and loss of spacing between vessels indicated that disorganized neovascularization occurred (Fig 2F and 2J). Fig 2K showed the quantitative analysis of Fig 2C–2J. The left panel shows the method for counting and measuring branch points (red asterisks) and vessel diameters (yellow line). These data consist of observed characteristics mentioned above.

### CoCl_2_ treatment leads to retinal vasculature leakage according to fluorescent dye injection

**Fig 3:**
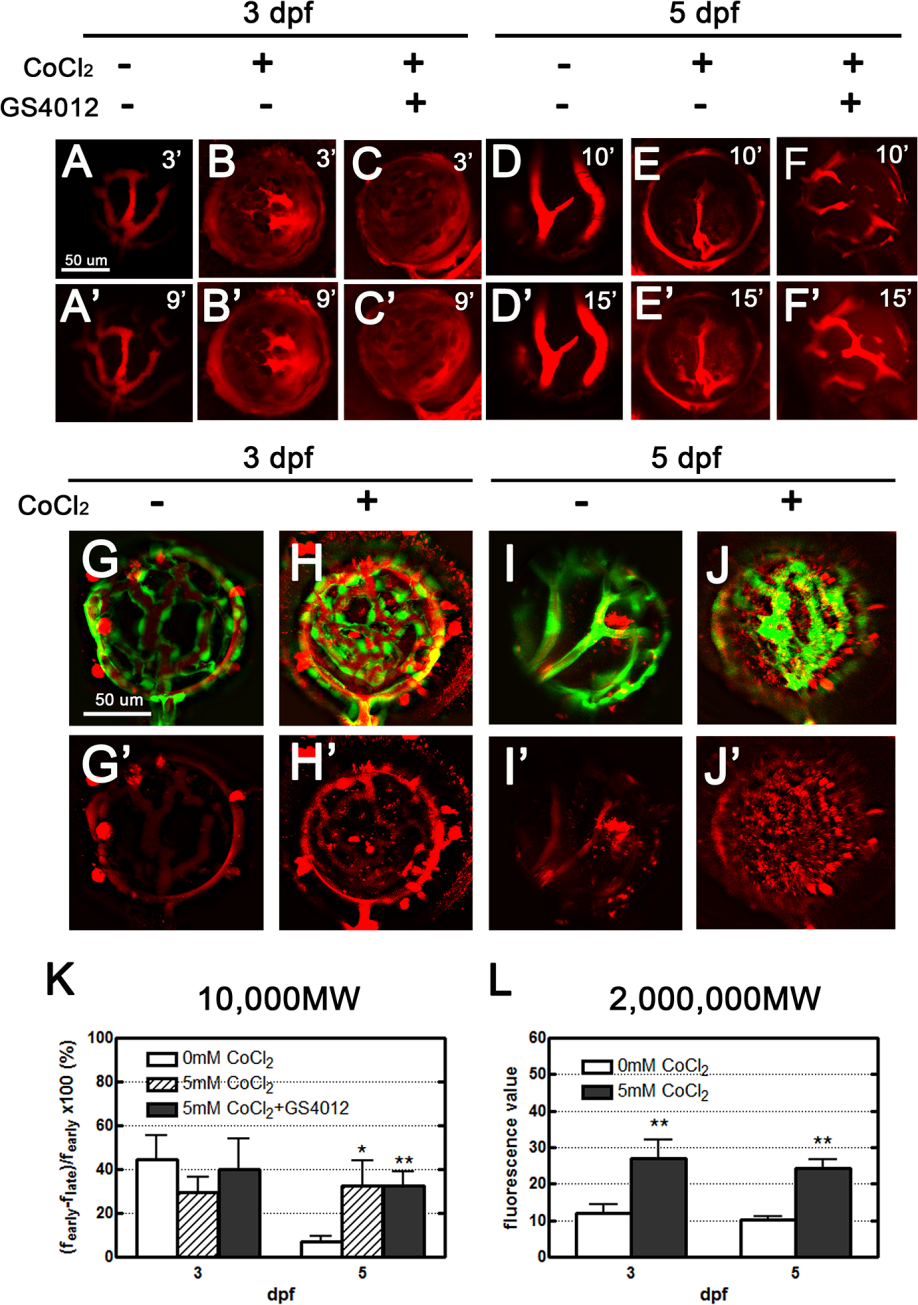
Leakage analyses of CoCl_2_-treated retinal vasculature performed using two types of fluorescent dyes: 10,000 MW dextran, and 2,000,000 MW TAMRA. (A–F’) Injected dextran (red) in the vessels became obscured, with apparent dextran leakage within 9 and 15 minutes in treated zebrafish embryos 3 and 5 dpf, respectively (B’, C’, E’, F’); however, in the control embryos, leakage was observed in the retinal vessels (A’). No leakage was apparent in the normal tight endothelium of 5-dpf embryos (D’). (G–J’) Upon injection, most of the TAMRA dye (red) was contained in the vasculature, and leakage was observed 24 hours later. Scant leakage occurred at the center of the control retina (G, I), whereas apparent leakage was observed in CoCl_**2**_-treated vasculatures (H, J). (K, L) The fluorescence values in 3- and 5-dpf embryos were quantified using Image J to show the dynamic changes of the 10,000 MW and 2,000,000 MW dyes. Data are presented as the mean ± standard deviation from three to five embryos.

Two types of fluorescent dyes, 10,000 MW Dextran and 2,000,000 MW TAMRA, were used in this study. In the mock control group, the 10,000 MW Dextran dye injection illustrated the clarity and simplicity of the organized vasculature with three main vessel branches, although slight leakage from intact healthy vessels occurred over time (Fig 3A and 3A’). The dye in CoCl_2_- and GS4012-cotreated zebrafish was not clearly defined. The increase in intensity of the fluorescent dye around the vasculature with time (9 minutes) was presumably caused by vessel leakage (Fig 3B and 3C’). Our quantified data similarly showed the futility of this 10,000 MW leakage tracer in 3-dpf retinal vessels (Fig 3K).

The effect of treatment on vessels at 5 dpf was superior to that at 3 dpf. The normal mock control group vasculature was clearly demarcated and simple, exhibiting no leakage at 10 minutes (Fig 3D). However, the vessels became obscured at 15 minutes, with some apparent leakage, but this was relatively low (Fig 3D’). Cotreatment with CoCl_2_ and GS4012 caused vessel narrowing, and traces of leakage were present between vessel branches (Fig 3E and 3F). The intensity of the red fluorescent leakage increased with each time interval and at 15 minutes, at which the intensity was the same as that of the fluorescence inside the vessels (Fig 3E’ and 3F’). According to the collected data, significant leakage consistent with the leakage characteristic of ROP occurred (Fig 3K).

Using a fluorescent dye injection greater than 2,000,000 MW, we clearly observed slight leaks in both 3-dpf and 5-dpf retinal vasculatures. Most of the TAMRA dye visibly remained within the retinal vessels in the control zebrafish (Fig 3G and 3I). The zebrafish treated with 5 mM CoCl_2_ exhibited marked leakage with drops into the surrounding vascular space, obscuring the view of the contorted vessels (Fig 3H and 3J). This increased leakage is consistent with that exhibited by ROP blood vessels (Fig 3L). The results indicated that the 2,000,000 MW TAMRA is the optimal dye for detecting leakage in the retinal vasculature.

According to the clinical description of ROP by the International Classification of Retinopathy of Prematurity [9], ROP can be classified by four parameters: location, stage, extent or clock hours of involvement, and the absence or presence of "plus disease". In our observations, the CoCl_2_-treated retina appeared extraretinal angiogenesis with a leakage (stage 3) and the blood vessels grew into the posterior zone of the retina defined as the circle with a radius extending from the optic nerve to double the distance to the macula (zone I). Additionally, significant level of vascular tortuosity observed at the posterior retinal arterioles reflects the increase of blood flow through the retina and indicates the complication (plus disease). These characters are consistent with the clinical scenario of ROP in zone I, stage 3 with plus disease [9, 23].

### Anti-VEGF treatments rescue zebrafish neovascularization

**Fig 4:**
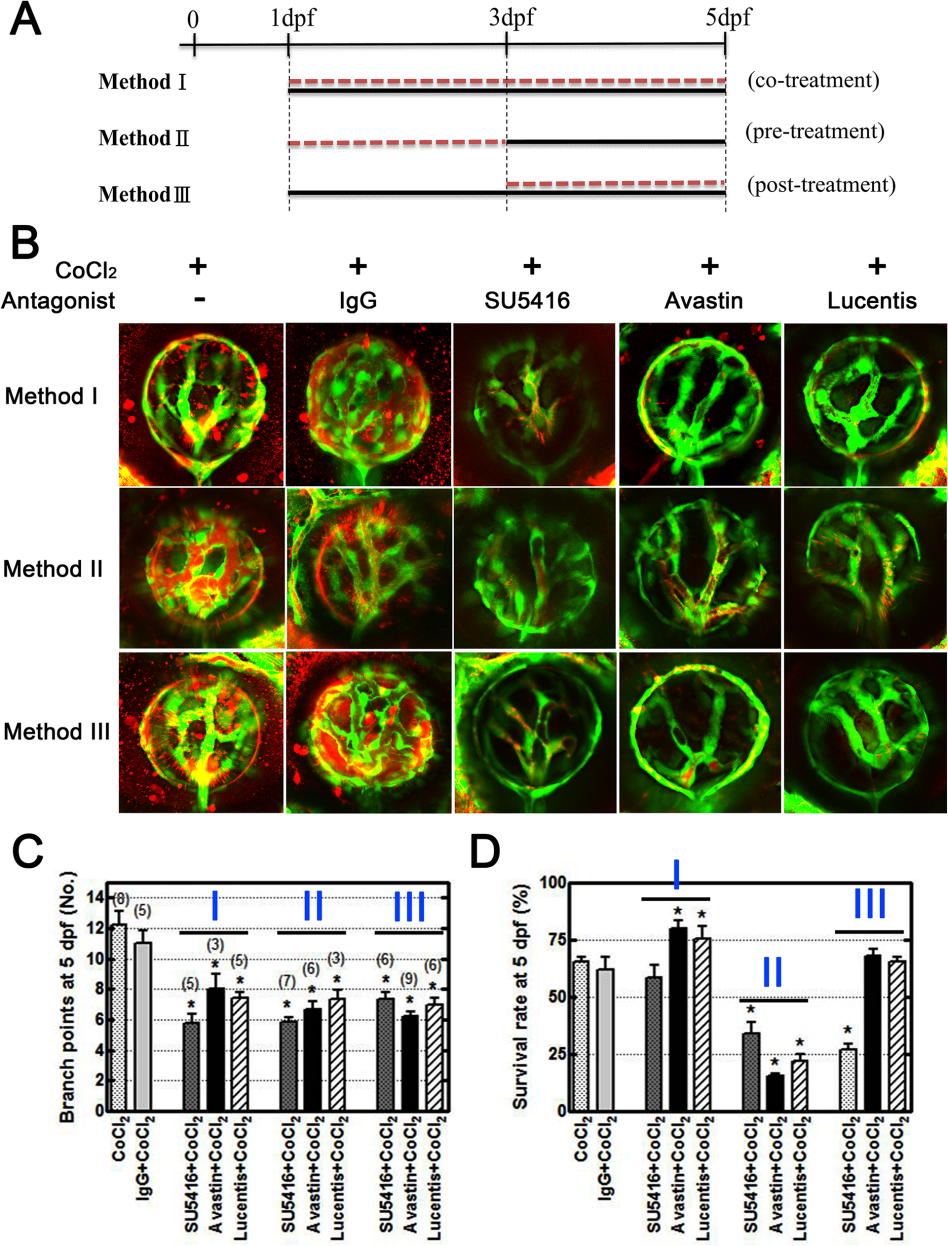
VEGF inhibitors rescue overangiogenesis and leakage in the CoCl_2_-induced hypoxic retinal vasculature. (A) Three conditions were designed to mimic the clinical situation and to examine the effects of treatment. Method I: Tg(*fli1a*:*EGFP*) embryos were treated with CoCl_**2**_ (black line) and the inhibitor (red dotted line) simultaneously from 1 dpf to 5 dpf. Method II: Prior to CoCl_**2**_ treatment (black line) at 3 dpf, we immersed embryos in the inhibitor (red dotted line) for 2 days. Method III: Following CoCl_**2**_treatment, the inhibitor was added to the solution at 3 dpf. All embryos were injected with TAMRA dye at 4 dpf, and their retinal vasculatures were subsequently observed at 5 dpf. (B) CoCl_**2**_-treated embryos showed TAMRA dye seepage (red) from the intraocular vessels (green). Similar leakages and excessive neovascularization were appeared in the control IgG-treated groups (CoCl_**2**_ and normal human control IgG cotreatment). Three candidate inhibitors, SU5416, bevacizumab, and ranibizumab, reversed the effect of CoCl_**2**_ on retinal vessels. (C) Statistical analyses of branch points showed significant rescue in inhibitor-treated embryos. The total numbers of embryos for analyses are indicated on the top of each bar. (D) However, the survival rates (four independent experiments, n = 35 in each) showed low toxicity under the Method I condition. Each bar represents the mean ± SEM of * (*p* < 0.0125) compared with the CoCl_**2**_ = 5 mM group.

To further validate our model, we used three candidate drugs, SU5416, bevacizumab, and ranibizumab, and observed vessel leakage at 5 dpf after fluorescent TAMRA dye injections. Bevacizumab and ranibizumab are anti-VEGF monoclonal antibodies used for retinal neovascularization and were used as positive controls to determine whether our disease model can be reversed. We conducted these experiments under three conditions: cotreatment (Method I), pretreatment (Method II), and posttreatment (Method III), which are illustrated in Fig 4A.

Under these three conditions, SU5416, bevacizumab, and ranibizumab treatment substantially prevented vessel leakage and branching compared with CoCl_2_ treatment alone or the control IgG-treated groups (Fig 4B). Statistical analyses of branch points in retinal vessels showed a significant decline while CoCl_2_-treated zebrafish were immersed in these candidate drugs, whereas the analysis showed no effect of the control IgG in CoCl2-treated fish (Fig 4C). We observed an increased survival ratio in zebrafish treated using Method I (Fig 4D). The results indicated that bevacizumab and ranibizumab are effective as expected, and Method I is optimal for preventing retinal neovascularization.

## Discussion

ROP is characterized by vasoproliferative and fibrotic changes resulting from hypoxia and the upregulation of proangiogenic mediators, such as HRE, HIF, and VEGF [8, 15]. For developing effective drug treatments, an animal model that replicates clinical neovascularization can provide an in-depth understanding of the underlying pathophysiological mechanism of retinopathy. Using a zebrafish retinopathy model is highly appropriate, because of the similarities shared by the retinal vasculatures of humans and zebrafish [27]. Previous neovascularization models of ROP have been created by inducing hypoxia [28] in zebrafish or other animals [8, 26, 41, 42]. Because the retinal morphology of adult zebrafish lacks the retinal vessel regression observed in humans, zebrafish embryos are appropriate for modeling the pathology. Zebrafish also exhibit the primitive angiogenesis branching from the central retinal artery observed in humans; this branching is the initial retinal vasculature associated with the lens and the basal lamina enclosing the retinal pericytes [27]. Our results support this phenomenon (Fig 1A).

Our qRT-PCR study demonstrated that CoCl_2_ is a potent chemical inducer of hypoxia and increases the gene expression of *vegfaa* and *vegfr2* (Fig 1E and 1F); these observations are consistent with the currently understood mechanism of ROP [8]. Hypoxia inhibits the degradation of HIF-1α protein [43]. Inactivated in normoxia, HIF-1α accumulates under hypoxic conditions and subsequently activates downstream *vegfaa*, producing VEGF and stimulating angiogenesis. Thus, by using both CoCl_2_ and GS4012, a VEGF inducer, our zebrafish model is consistent with this proposed pathway. Our mRNA analysis indicated that *vegfaa* and *vegfr2* expression occurs at 3 dpf, with an approximate two-fold increase under a chemical hypoxia condition (Fig 1E). A previous study showed that VEGF expression induced the feedback mechanism of VEGFR2 through the DII4-Notch cascade [44]. In retina tip cells, VEGF induced Sema3E–Plexin-D1 signaling and downregulated VEGF-induced DII4 expression [40]. Notch activity subsequently decreased in stalk cells and upregulated the level of VEGFR2, leading to vascular sprouting [44]. In our study, CoCl_2_ treatment induced an exuberant developed capillary network in the retinal vessels (Figs 1B and 2D) Therefore, the activation of Sema3E–Plexin-D1 signaling in the retinal vessels may have occurred and promoted VEGFR2 expression (Fig 1F, 1G and 1H). At 5 dpf, the decreased retinal vessel sprouts reduced Plxnd1 expression and then downregulated VEGFR2 expression (Figs 1F, 1G, 1H and 2H).

Using Tg(*fli1a*:*egfp*) zebrafish enabled us to visualize the vasculature without using additional staining techniques. ROP is characterized by rapid pathological neovascularization, leaky dysfunctional vessels, and fibrovascular proliferation under hypoxic conditions [8]. Confocal analysis showed that our hypoxia-induced model exhibited an increased number of vascular branch points and sprouts, tortuosity of vessel arrangement, and vasoconstriction in the retinal vessels (Fig 2). Fluorescent dye injection analysis showed that both the 10,000 MW Dextran and 2,000,000 MW TAMRA dyes leaked through the retinal vessels (Fig 3). Although vessel leakage occurred in the mock control, more extensive leakage occurred in our hypoxia model, obscuring the vessel clarity under confocal analysis. This finding indicates that tight junctions between endothelial cells were poorly formed, resulting in leakage of all molecules, regardless of size.

SU5416 is a competitive inhibitor of Flk-1/KDR and VEGFR-2 [1], which blocks the effects of VEGF, inhibiting angiogenesis and neovascularization in our model. Adding SU5416 alone inhibited vascular growth in fish with retinal vessels at 3 dpf, yielding an appearance consistent with that of the 1-dpf mock control group zebrafish (S2A Fig). Development was suppressed to a point where oxygen deficiency and undernourishment of peripheral tissues caused the embryos to no longer be viable. Subsequently, we observed high mortality after 3 dpf, illustrating the importance of VEGF in angiogenesis in the retina. Treatment with SU5416 after hypoxia was induced reduced the extensive branching and tortuosity that we observed in our model (Fig 4B). Cotreatment with CoCl_2_ and SU5416 provided a favorable organized vasculature at 3 dpf, with vascular leakage similar to that of the mock control (S2B Fig). However, at 5 dpf, this was no longer the case because SU5416 suppressed any further growth and regressed retinal vessels. In our fluorescent dye analysis, no dye was present within the retinal vasculature, although the inner optic circle continued to leak. This is most likely due to continual inhibition from SU5416, causing the retinal vessels to become nonfunctional.

To mimic ROP treatment methods, we designed three conditions in this study and compared their therapy efficiency (Fig 4A). Method I involves cotreating embryos with candidate drugs and CoCl_2_. Method II entails pretreating embryos with the candidate drugs 2 days prior to CoCl_2_ exposure and was used to evaluate the efficacy of primary prophylactic treatment. Method III involves administering antagonist posttreatment following ROP establishment; 3-dpf embryos are treated until 5 dpf for confocal analysis. We followed the current protocol for clinical practice. At 5 dpf, we found the three methods to be effective in reducing the number of branch points and sprouts, vasoconstriction, and the disorganized architecture that was induced under hypoxia (Fig 4B and 4C). These changes were more regular than those of the CoCl_2_-treated embryos, with the distinct vasculature and vessel leakage similar to that of the mock control group. However, the survival rates showed high toxic effects in zebrafish subjected to Method II and low toxic effects in zebrafish subjected to Method I (Fig 4D), indicating that Method I is the optimal ROP treatment. Additional studies of high animal models are necessary to confirm this phenomenon.

Several clinical studies have reported using bevacizumab and ranibizumab for treating ROP [23, 45–48]; specifically, studies have used bevacizumab in monotherapy [48, 49], in combination with laser therapy [50, 51], as rescue therapy after failed laser photocoagulation [52], and in combination with or prior to vitrectomy for ROP [52]. The Bevacizumab Eliminates the Angiogenic Threat of Retinopathy of Prematurity study [50], which compared intravitreal administration of bevacizumab with laser therapy, showed that bevacizumab improved outcomes only for zone 1, stage 3 ROP with plus disease; another study obtained similar findings [50, 51]. The results suggested that using anti-VEGF agents to reduce severe ROP might be promising; however, additional studies regarding drug doses and their timing, the type of anti-VEGF agent, and safety are warranted. The pathologies and treatments of our model are equivalent to those of zone 1, stage 3 human ROP, and our model provides merits for ROP study

Bevacizumab and ranibizumab are recombinant humanized monoclonal IgG antibodies that blocks angiogenesis by inhibiting VEGF-A. Both of them contain human framework regions and the complementarity-determining regions of a murine antibody that binds to VEGF. However, Alvarez et al. reported bevacizumab is not anti-angiogenic in zebrafish [39], and Chimote et al. indicated the absence of anti-angiogenetic efficacy with bevacizumab resulted from extremely variable results or the inability of monoclonal antibodies to permeate sufficiently to have an effect on the SIVs [53]. Therefore, comparing the sequence of zebrafish VEGF protein with that of human VEGF protein, we found that 75% of the protein sequence in zebrafish VEGFAA is similar to that of human VEGFA165 isoform. We believe that it is possible for bevacizumab and ranibizumab to inhibit human and zebrafish VEGF. In our study, we used human control IgG as a control to prove the specific interaction with antibodies and observed the anti-angiogenic effects of these antibodies on the retinal neovascularization in zebrafish (Fig 4B).

We observed that blood cells obviously accumulated in the tail after CoCl_2_ treatment at 1 dpf when the blood cells began circulating on the yolk ball [54], indicating that CoCl_2_ induced abnormal angiogenesis and development of the vasculature, including the SIV and retinal vessels. The fact that the SIV plays a primary role in yolk absorption and is fed by venous blood from the caudal vein [55] explains the slight development delay of CoCl_2_-treated embryos in our study. Furthermore, the number of SIV branches in CoCl_2_-treated embryos decreased with the number of certain sprouted vessels (red arrows, Fig 1B) compared with those in the control. Then, we identified the embryos according to branch number in the SIV at 3 dpf and the inflation of the swim bladder at 5 dpf as previously described [54, 56]. Reinardy et al. indicated that the lethal concentration for 50% mortality in larval zebrafish exposed (96 h) to 0–50 mg/L of Co was 35.3 ± 1.1 (95% confidence interval) mg/L [57]. Our study showed the same mortality rate (50%) at 5 mM and 10 mM concentrations, although the concentrations we used were higher than those used by Reinardy et al. At this mortality rate, we conducted our study of residual live larvae. Kajimura and Yu used the concentrations of CoCl_2_ used in the present study to induce HIF-1α expression [58, 59]. Therefore, our strategy for collecting live larvae and measuring the expression of neovascularization-related genes such as *vegfaa* and *vegfr2* in CoCl_2_-treated embryos is reasonable, although the expression level of *vegf* may come from extra-ocular tissues.

Our findings show that the retinal vasculature of the zebrafish embryo is similar to that of humans in response to hypoxia, and the progression of pathological angiogenesis is consistent with that of ROP. Using a zebrafish model enables rapid, efficient, candidate-drug screening without the high cost or maintenance that a mammalian model demands. A simple zebrafish model of ROP that replicates the clinical scenario can provide a clearer understanding of the mechanism of ROP development and facilitate research into new treatment methods.

## Supporting Information

S1 Fig Early development of retinal neovascularization in zebrafish. (A–C) Confocol images of GFP expression in the eye of Tg(*fli1a*:*EGFP*) zebrafish. (A) DAPI staining (blue) was used to orient the ocular blood vessels at 6 dpf. (B) In the surface vasculature, blood enters through the nasal vessel (nrv) and exits through the dorsal (drv) and ventral (vrv) vessels. Blood from the retinal vessels flows through the annular collection duct (asterisks) into the surface vessels. (C) Lateral view of the retinal vessel network. The retinal artery or its presumptive primordium is indicated by the red arrowhead. (D) Retinal vessels at 6 dpf. (E) At 1 dpf, uniform growth of the retinal vessels from the central retinal artery was observed. (F) The vessels assumed a cup shape at 2 dpf. (G) The retinal vessels increased in number at 3 dpf. (H) Vessel branching was apparent and the vascular architecture complexity increased in the retina 4 dpf. (I) Vessel branching and organization further developed, as indicated by the formation of numerous branch points and sprouts at 5 dpf.
(TIF)

S2 Fig Effect of SU5416 on retinal vessels at 3 dpf. (A) SU5416 inhibited the development of the retinal vessels. (B) SU5416 reversed the effect of CoCl_2_ on the retinal vessels and prevented the leakage of TAMRA dye through the vessels.
(TIF)
