# Supplementary figures and images for: Hypoxia-Induced Retinal Neovascularization in Zebrafish Embryos: A Potential Model of Retinopathy of Prematurity
Source: PLoS One. 2015 May 15;10(5):e0126750. doi: 10.1371/journal.pone.0126750 (PMC4433197; doi:10.1371/journal.pone.0126750)

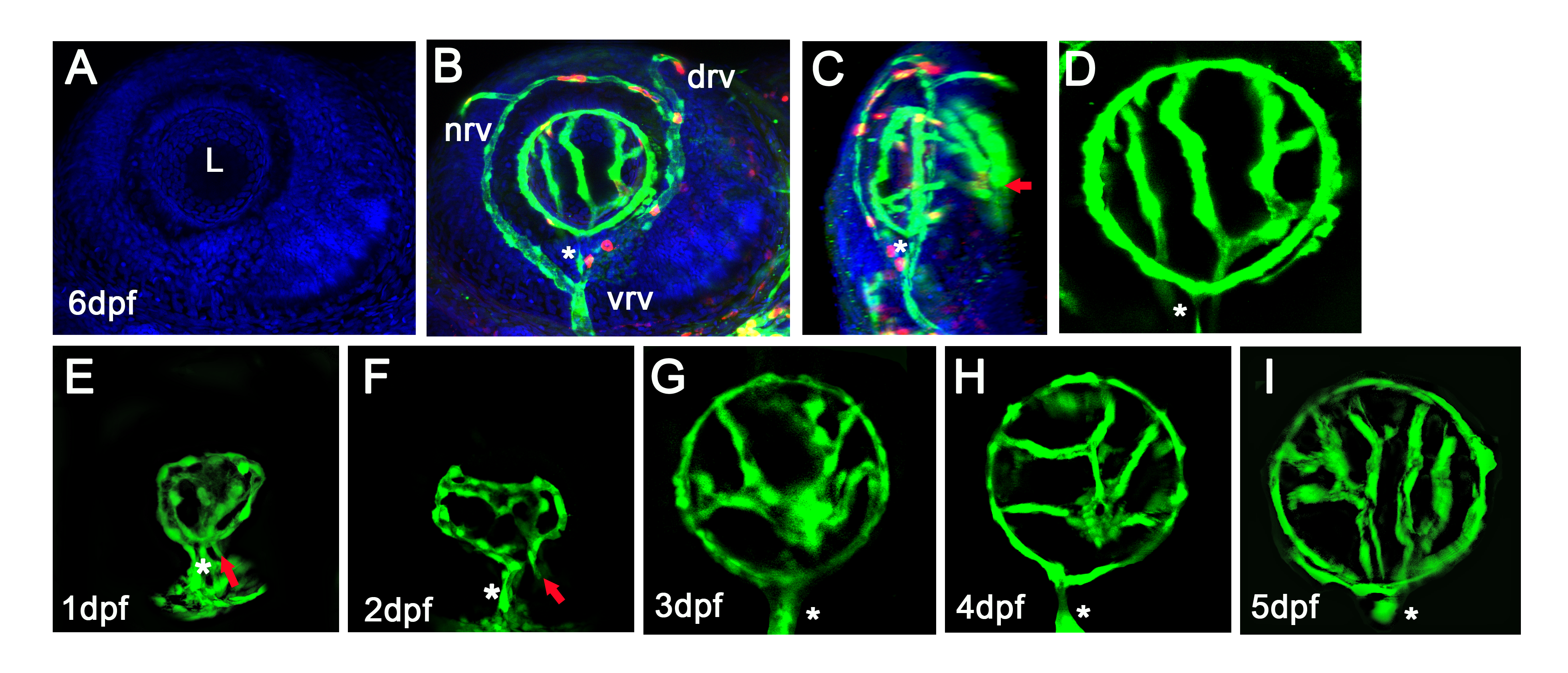

Supplement: S1 Fig — (A–C) Confocol images of GFP expression in the eye of Tg(fli1a:EGFP) zebrafish. (A) DAPI staining (blue) was used to orient the ocular blood vessels at 6 dpf. (B) In the surface vasculature, blood enters through the nasal vessel (nrv) and exits through the dorsal (drv) and ventral (vrv) vessels. Blood from the retinal vessels flows through the annular collection duct (asterisks) into the surface vessels. (C) Lateral view of the retinal vessel network. The retinal artery or its presumptive primordium is indicated by the red arrowhead. (D) Retinal vessels at 6 dpf. (E) At 1 dpf, uniform growth of the retinal vessels from the central retinal artery was observed. (F) The vessels assumed a cup shape at 2 dpf. (G) The retinal vessels increased in number at 3 dpf. (H) Vessel branching was apparent and the vascular architecture complexity increased in the retina 4 dpf. (I) Vessel branching and organization further developed, as indicated by the formation of numerous branch points and sprouts at 5 dpf. (TIF) [file pone.0126750.s001.tif]

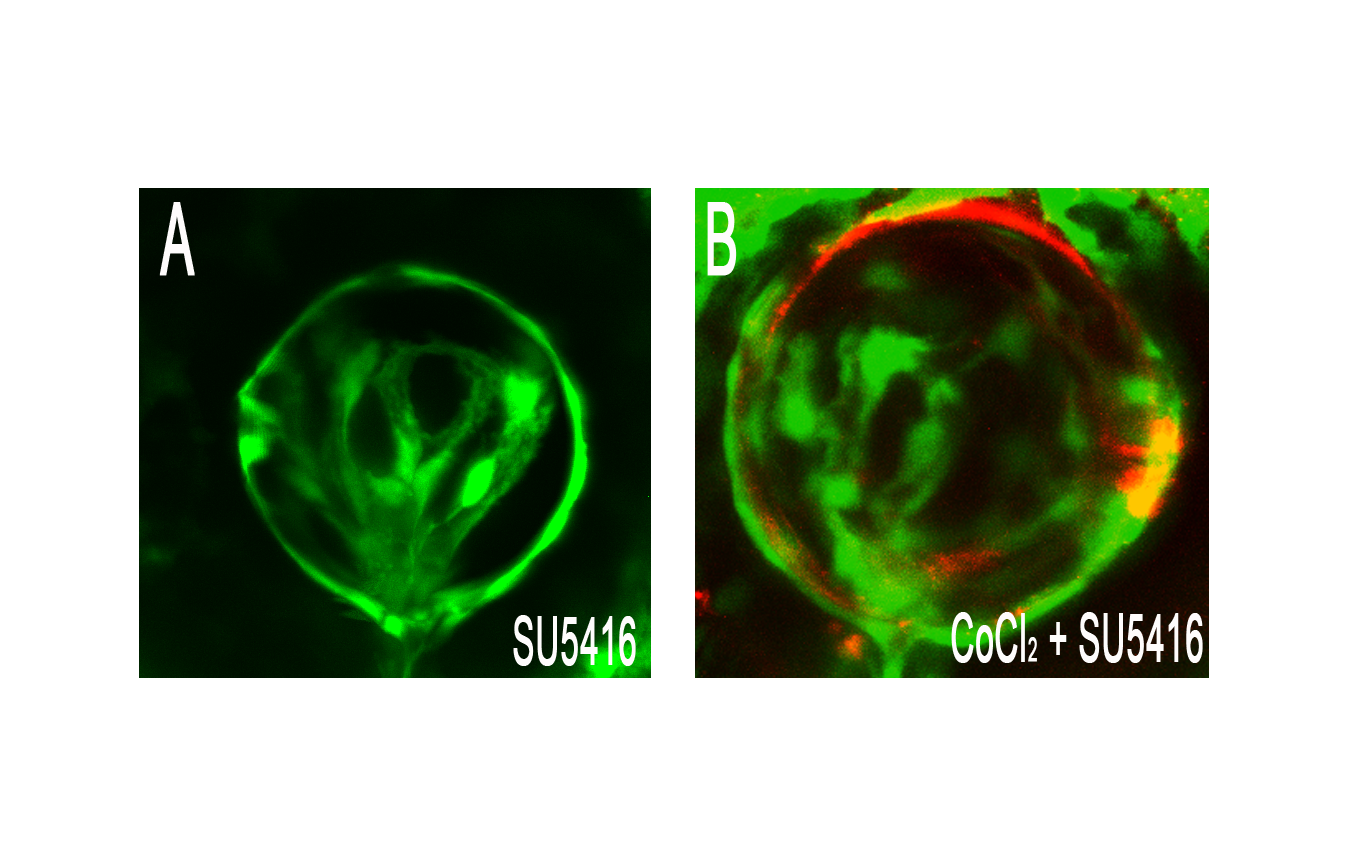

Supplement: S2 Fig — (A) SU5416 inhibited the development of the retinal vessels. (B) SU5416 reversed the effect of CoCl2 on the retinal vessels and prevented the leakage of TAMRA dye through the vessels. (TIF) [file pone.0126750.s002.tif]
